# Atypical Manifestation of VZV Infection in a Vaccinated Immunocompetent Adult

**DOI:** 10.1155/2022/5626670

**Published:** 2022-11-11

**Authors:** Deesha Bhojwani, Mohamed Zakarya, Suha Abu Khalaf

**Affiliations:** University of Missouri, One Hospital Dr DC043.00, Columbia, MO 65212, USA

## Abstract

**Introduction:**

Aseptic meningitis can occur from different types of infections of which viral etiologies are the most common. Varicella zoster virus (VZV) nowadays is considered a familiar entity of aseptic meningitis. However, it is usually reported in immunocompromised patients. For cases of VZV meningitis that are observed, a rash has been noted before the onset of meningitis or sometimes after it. *Clinical Case.* We present an uncommon case of VZV meningitis in an 18-year-old immunocompetent male who did not have a rash on presentation and did not develop one during his course either. Cerebrospinal fluid showed lymphocyte-predominant leukocytosis and elevated protein with normal glucose suggestive of aseptic meningitis. Cerebrospinal fluid polymerase chain reaction (CSF PCR) was positive for VZV; cerebrospinal fluid cultures and blood cultures were negative. The patient had complete resolution of symptoms with no complications on intravenous acyclovir therapy and was discharged home on oral valacyclovir therapy.

**Conclusion:**

It is important to consider varicella zoster virus as an etiology of aseptic meningitis as clinical presentation can be without a vesicular rash and in immunocompetent patients.

## 1. Introduction

VZV is a human alpha herpes virus that can lead to primary infection which most commonly manifests as varicella/chickenpox. Following primary infection, it becomes latent in ganglionic neurons and adrenal glands. In the setting of immune deficiency, it reactivates to cause herpes zoster (shingles) [1, 2]. Primary varicella infections as well as reactivated infections can lead to central nervous system (CNS) manifestations such as meningitis, encephalitis, or meningoencephalitis in immunocompetent as well as immunocompromised hosts. CNS manifestations are more severe in immunocompromised individuals [3]. Most cases of meningitis/encephalitis due to varicella zoster virus are associated with a rash that develops either at the onset of symptoms or later in the disease course [3]. We present an uncommon case of an 18-year-old immunocompetent male patient who presented with severe headache and fever. CSF studies were suggestive of aseptic meningitis, and the diagnosis of varicella zoster virus (VZV) meningitis was confirmed on CSF PCR. The patient, however, did not develop a rash during the disease course. It is an uncommon presentation of VZV meningitis in an immunocompetent adult without the development of a rash during the clinical course of the disease.

## 2. Case Presentation

### 2.1. Patient Information

An 18-year-old man with no significant medical history presented to our hospital with progressively worsening headache and persistent fever of one-day duration. The patient described the headache as the worst headache of his life and was squeezing, band-like in nature. It was associated with photophobia and neck stiffness. The patient also had associated body aches, fatigue, nausea, and multiple episodes of vomiting. His only sick contact was four days prior to his presentation with his friend who had influenza.

### 2.2. Relevant Past Interventions/History

The patient was vaccinated against varicella at the age of five. He denied any history of febrile exanthems or cold sores in childhood.

### 2.3. Clinical Findings

The patient was noted to be febrile and tachycardic (Table 1). Nuchal rigidity was present on examination. No mucosal or cutaneous rash was noted on physical examination (Table 2).

### 2.4. Diagnostic Assessment

The patient had granulocyte-predominant leukocytosis on presentation (Table 3). CT head was performed outside the hospital which was negative for any intracranial abnormalities. Cerebrospinal fluid studies showed lymphocyte-predominant leukocytosis, elevated protein, and normal CSF glucose (Table 3). Hence, CSF studies were suggestive of aseptic meningitis. CSF meningitis polymerase chain reaction was reactive for varicella zoster virus which confirmed the diagnosis of varicella zoster virus meningitis. Cerebrospinal fluid culture, blood culture, and urine culture remained negative. Human immunodeficiency virus antigen-antibody testing was nonreactive. Herpes simplex virus 1 and 2 polymerase chain reactions were negative.

### 2.5. Therapeutic Interventions

The patient was started empirically on intravenous (IV) vancomycin (1.5 grams three times daily as per weight-based dosing of ∼12 mg/kg/dose with goal vancomycin trough of 15–20 mcg/ml) and ceftriaxone (two grams every twelve hours) prior to lumbar puncture. IV acyclovir (800 mg three times daily) was added to empirical antimicrobial therapy when CSF studies suggested aseptic meningitis. After CSF PCR tested positive for VZV, vancomycin and ceftriaxone were discontinued. The patient was continued on IV acyclovir (10 mg/kg of ideal body weight) for a total of five days. The patient was discharged on oral valacyclovir (2 g every six hours) for nine days to complete a total of fourteen days of antiviral therapy. A timeline of the patient's clinical course is given in Table 4.

### 2.6. Follow-Up and Outcomes

The patient's clinical condition started to improve after 24 hours of empiric intravenous vancomycin, ceftriaxone, and acyclovir initiation. His clinical condition improved remarkably with the resolution of his symptoms within forty-eight hours of IV acyclovir therapy. The patient was advised to have a close follow-up with a primary care provider after the completion of his antiviral therapy. No rash was noted on examination during his five days of hospitalization. Per patient's mother and primary care provider, no rash developed within one-month postdischarge.

## 3. Discussion

VZV is associated with numerous manifestations ranging from chickenpox, herpes zoster to CNS manifestations such as meningitis, encephalitis, meningoencephalitis, myelopathy, and cerebellitis [2]. The proposed mechanism of spread of VZV in primary CNS disease is hematogenous spread which involves vesicular trafficking from trigeminal ganglion to cerebral arteries via the ophthalmic branch [3]. Although enterovirus is the most common cause of aseptic meningitis, especially in childhood, VZV is the second most common cause, with 4.4–11% of suspected meningitis cases testing positive for VZV DNA by PCR [3]. Earlier, VZV was not as routinely tested on CSF as enterovirus and HSV 1, 2. However, given better diagnostic studies and wide availability of multiplex PCR testing, the incidence of VZV meningitis has been rising [4].

Primary varicella infections as well as reactivated infections can lead to CNS manifestations in immunocompromised patients and uncommonly in immunocompetent patients. Meningitis and encephalitis are the most common CNS manifestations in adults. VZV infections are usually associated with a rash; however, it is essential to note that cases without a rash have been reported in literature. In primary varicella as well as herpes zoster, CNS manifestations can precede the onset of rash, and hence, even if the rash is absent initially during clinical presentation, it can develop later in the disease course [3]. Additionally, there have been atypical presentations without a rash during the entire clinical course. Even severe manifestation of VZV infection, visceral VZV has been reported in an immunocompromised patient without a rash [5].

There are a total of fifty-five cases of VZV meningitis reported in immunocompetent patients from 1997 to 2022 (Table 5). Of the fifty-five reported cases, eighteen presented without a rash which suggests that our case presentation is uncommon and atypical [6]. Two case reports were reviewed which describe varicella zoster virus meningitis in immunocompetent healthy children at age eight and fourteen who have presented without a rash. The patients had a history of prior varicella infection in both case reports [7, 8]. Ibrahim et al. also reported herpes zoster meningitis in immunocompetent patients without an eruption of a shingles rash [9]. An interesting case has been reported by Spernovasilis et al. of a 36-year-old immunocompetent male with VZV meningitis who presented without a rash [10].

A reduction in neurological morbidity has been observed with antiviral therapy due to which it is essential to diagnose VZV meningitis promptly [4]. Several antiviral agents are effective against VZV; however, acyclovir is the drug of choice given its higher efficacy with lower adverse events. Given the poor bioavailability of oral acyclovir, intravenous (IV) acyclovir is the preferred route [3, 11]. IV acyclovir offers rapid clinical improvement in addition to reduction in long-term complications [12]. Gnoni et al. reported a 29-year-old immunocompetent patient with VZV meningitis that was successfully treated with IV acyclovir for 2 days followed by oral valacyclovir [13]. Valacyclovir is the prodrug of acyclovir that offers three to four times higher oral bioavailability. A dose of two grams every six hours of oral valacyclovir has been found to be equivalent to 10 mg/kg of IV acyclovir every eight hours based on plasma concentration [3, 11].

## 4. Conclusion

This case provides salient evidence that VZV meningitis can present atypically in young immunocompetent adults. A high index of suspicion is necessary to be able to promptly diagnose and treat this condition before neurological complications ensue. When a CSF infection such as meningitis or encephalitis is suspected, CSF should be routinely tested for VZV along with HSV 1 and 2 [14].

## Figures and Tables

**Table 1:** Physical examination findings.

General	The patient was lying in a dark room with the eyes closed and was distressed due to pain
Head	Atraumatic and normocephalic
Eyes	Anicteric sclera, no conjunctival pallor, or injection
Neck	Nuchal rigidity noted
Respiratory	Unlabored, symmetric chest rise, on room air, no wheezing noted, and chest clear to auscultation bilaterally
Cardiovascular	Tachycardic, patient in sinus rhythm, and no murmurs appreciated on auscultation
Abdomen	Soft, nontender, nondistended, and no hepatosplenomegaly
Neurological	Alert, attentive, and oriented to time; place person, and situation; cranial nerves two to twelve grossly intact; no loss of muscle bulk or tone; sensations to light touch symmetrical and intact in all extremities; strength 5/5 in all extremities, reflexes 2+ at biceps, triceps, knees, and ankles; normal gait; coordination tested by rapid alternating movements intact in all extremities
Skin	Warm, dry, and no mucocutaneous rash noted

**Table 2:** Vital signs.

Temperature (degree Celsius)	38.4 (location temporal)
Heart rate (beats per minute)	119
Blood pressure (mmHg)	117/59
Respiratory rate (per minute)	20
Oxygen saturation (%)	96% on room air

**Table 3:** Laboratory findings.

Leukocyte count (per liter)	10.92 × 10 (9)
Hemoglobin (g/dl)	15.2
Platelet count (per liter)	331 × 10 (9)
CSF white blood cell count (per high power field)	109 (critical high >30)
CSF lymphocyte (%)	55%
CSF macrophages/monocytes (%)	4%
CSF neutrophils (%)	41%
CSF protein (mg/dl)	89 (normal 15–45 mg/dl)
CSF glucose (mg/dl)	56 (normal 40–70 mg/dl)
Serum glucose (mg/dl)	118

**Table 4:** Timeline of clinical events.

Day 0	Fatigue, headache, nausea, vomiting, and neck stiffness
Day 1	The patient was started on IV vancomycin and ceftriaxone. CT head was unremarkable. A lumbar puncture was performed in the emergency room which was suggestive of aseptic meningitis. IV acyclovir started.
Day 2	Patient's headache, nuchal rigidity, nausea, and vomiting significantly improved. Afebrile. CSF PCR positive for VZV. IV vancomycin and ceftriaxone discontinued. The patient continued to be on IV acyclovir
Day 3	Near complete resolution of symptoms. Afebrile. IV acyclovir continued
Day 4	Asymptomatic, afebrile. On IV acyclovir
Day 5	Completed five days of IV acyclovir and discharged home on oral valacyclovir
Day 16	Completed nine days of oral valacyclovir; followed up with primary care provider

**Table 5:** 

Author	Patient's age	Rash	Year
Kerr C.	39	Present	2022
Maruki T.	71	Present	2022
Landa E.	69	Present	2022
Yun S.	18	Absent	2022
Lagrine M.	14	Absent	2021
Jia S.	30	Absent	2021
Khalil S.	Not available	Present	2021
Raghunathan	32	Absent	2021
Platanki	56	Information not available	2021
Bateman	36	Present	2021
Bhandari	60	Present	2021
Bhandari	29	Present	2021
Bhandari	22	Present	2021
Sahra S.	31	Present	2021
Imani	32	Present	2020
Ashour A.	39	Absent	2020
Alataby	40	Present	2020
Faluk	45	Present	2020
Sohal	40	Present	2020
Davidson N.	28	Present	2019
Chan	15	Present	2019
Fadhel	44	Absent	2019
Khaliq	28	Present	2018
Ryu	11	Present	2019
Spernovasilis	36	Absent	2018
Suri	24	Absent	2018
Gnoni	29	Present	2018
Kim	16	Present	2017
Kim	8	Present	2017
Itoh	12	Present	2017
Itoh	12	Present	2017
Ganesan	70	Present	2016
Ibrahim	15	Absent	2015
Abe	57	Absent	2015
Sanguankeo	51	Present	2015
Pasedag	18	Absent	2014
El-Safadi	39	Absent	2014
Esposito	14	Present	2013
Goyal	27	Present	2013
Mantero	17	Absent	2013
Kangath	30	Present	2013
Han	7	Present	2011
Klein	53	Absent	2010
Spiegel	14	Absent	2010
Pena	11	Present	2009
Iyer	9	Present	2009
Habib	26	Absent	2009
Leahy	14	Absent	2008
Mpaka	66	Absent	2008
Frantzidou	72	Information not available	2008
Frantzidou	15	Information not available	2008
Haargaard	64	Present	2008
Pirounaki	27	Present	2007
Hartzell	21	Present	2006
Moriuchi	37	Present	1997

## Data Availability

The data used to support the findings of this study are included within the article.
